# miR-21, Mediator, and Potential Therapeutic Target in the Cardiorenal Syndrome

**DOI:** 10.3389/fphar.2020.00726

**Published:** 2020-05-15

**Authors:** Cheng-Kai Huang, Christian Bär, Thomas Thum

**Affiliations:** ^1^ Institute of Molecular and Translational Therapeutic Strategies, Hannover Medical School, Hannover, Germany; ^2^ REBIRTH Center for Translational Regenerative Medicine, Hannover Medical School, Hannover, Germany

**Keywords:** microRNA, non-coding RNA (ncRNA), antisense-oligonucleotides, cardiorenal syndrome (CRS), miR-21, miR-21 inhibitor

## Abstract

Oligonucleotide-based therapies are currently gaining attention as a new treatment option for relatively rare as well as common diseases such as cardiovascular disease. With the remarkable progression of new sequencing technologies, a further step towards personalized precision medicine to target a disease at a molecular level was taken. Such therapies may employ antisense oligonucleotides to modulate the expression of both protein coding and non-coding RNAs, such as microRNAs. The cardiorenal syndrome (CRS) is a complex and severe clinical condition where heart and renal dysfunction mutually affect one another. The underlying mechanisms remain largely unknown and current treatments of CRS are mainly supportive therapies which slow down the progression of the disease, but hardly improve the condition. The small non-coding RNA, microRNA-21 (miR-21), is dysregulated in various heart and kidney diseases and has been repeatedly suggested as therapeutic target for the treatment of CRS. Impressive preclinical results have been achieved by an antisense oligonucleotide-based therapy to effectively block the pro-fibrotic traits of miR-21. Since microRNA-mediated pathways are generally very well-conserved, there is considerable commercial interest with regards to clinical translation. In this review, we will summarize the role of miR-21 within the heart–kidney axis and discuss the advantages and pitfalls of miR-21 targeting therapeutic strategies in CRS.

## Cardiorenal Syndrome And Current Therapies

**Figure 1:**
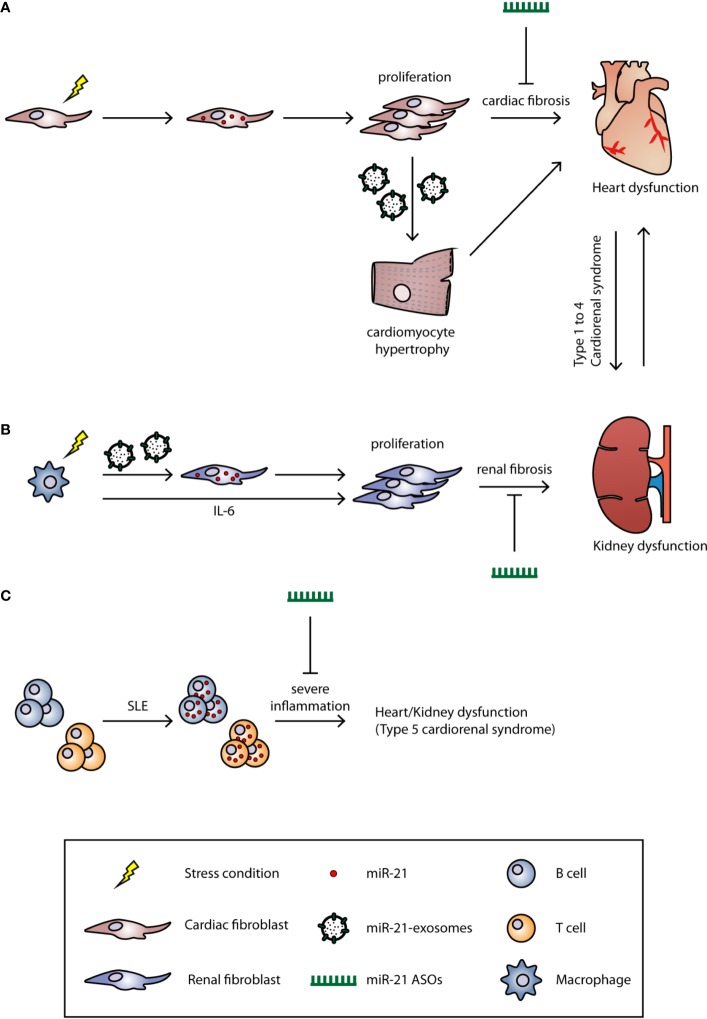
The role of miR-21 in cardiorenal syndrome. The underlying mechanisms of CRS are still unclear, and the current classification system for CRS is based on the clinical phenotype. Based on the primary organ dysfunction, CRS can be categorized into five subtypes. Under certain stress conditions (hypertension, LPS, or inflammation), the miR-21 is upregulated in **(A)** cardiac or **(B)** renal fibroblast and induces fibrosis. MiR-21 can also be transported by microvesicles to other cell types to induce fibrosis. **(C)** In SLE, the miR-21 is upregulated in both B and T cells which induces strong inflammation. Treatment of miR-21 ASOs could significantly alleviate the fibrosis and, therefore, improve the cardiac and renal function.

Cardiorenal syndrome (CRS) is a condition that describes interdependent disease conditions where a primary dysfunctional organ causes dysfunction in a secondary organ, in this case, the heart and the kidneys (Shah and Greaves, 2010; Braam et al., 2014). In an attempt to classify CRS, Ronco et al. defined five different subtypes in 2008 (Ronco et al., 2008). In CRS types 1 and 2, acute heart injury (AHI) and chronic heart failure (CHF) precede acute kidney injury (AKI) and chronic kidney disease (CKD), respectively. Subtypes 3 and 4 showed the opposite with the kidney being the primary organ affected through AKI and CKD, leading to AHI and CHF. CRS type 5 describes both heart and kidney dysfunction that coexist as the result of other systemic diseases (**Figure 1**). Since this classification system was proposed 12 years ago, it lacks a more precise definition owing to the complex interaction between the heart and the kidneys (Ronco et al., 2008). The heart is the most important organ in the circulatory system and distributes nutrients and oxygen to other organs, including the kidney. The kidney is the major organ responsible for regulating salt and water balance of the whole body. For decades, it was believed that impaired cardiac function leads to low cardiac output and therefore decreases the kidney glomerular filtration rate (GFR). This renal dysfunction then results in increased fluid retention which in turn leads to increased cardiac pre- and afterload (Ronco et al., 2008). However, it has been demonstrated that improving cardiac function in type 1 and type 2 CRS patients did not improve renal function (Nohria et al., 2008). This discrepancy between theory and clinical observation underlines the notion that the underlying mechanisms of CRS still remain largely unknown. Nevertheless, the principle therapy for CRS, especially type 1 and type 2, is aimed at improving cardiac function. For example, using diuretics to reduce fluid overload and blood pressure is a common treatment option for CRS. However, such anti-hypertensive treatments require close monitoring since hypotension and renal hypoperfusion can lead to worsening of kidney function (Hudson et al., 2003). Other treatments, such as angiotensin converting enzyme (ACE) inhibitors, vasodilators, and inotropic drugs, are also commonly used for treatment (House, 2013). In type 3 and type 4 CRS, inflammation caused by AKI or CKD leads to elevated cytokine expression and leukocyte infiltration in the heart (Bryant et al., 1998; Daemen et al., 1999; Kelly, 2003; Akcay et al., 2009; Chuppa et al., 2018). This may trigger cardiomyocyte apoptosis which in turn contributes to impaired cardiac function. In addition, fluid and electrolyte imbalance and uremia toxicity are also recognized as drivers of type 3 and type 4 CRS. Currently, there are no effective therapies specific for types 3 and 4 CRS and the only available option to use diuretics to improve fluid removal from the circulation (Chuasuwan and Kellum, 2012; Clementi et al., 2013). Systemic diseases, such as sepsis and diabetes mellitus (DM), are the main reason for type 5 CRS. Like other types of CRS, systemic diseases induce strong inflammatory responses which can impair both cardiac and renal function simultaneously. Therefore, the first therapeutic option is to treat the detrimental inflammation. This includes the use of antibiotics and inotropic drugs for sepsis patients, whereas insulin, to lower blood glucose levels, is given to DM patients (Soni et al., 2012).

## MiRNAs and Their Role In Cardiorenal Syndrome

Owing to the rapid development of next-generation sequencing (NGS) technologies over the last decade, it became evident that vast parts of our genome are actively transcribed into non-coding RNAs (ncRNAs), while the protein coding transcripts, i.e., messenger RNAs (mRNAs), are transcribed by only 1% to 2% of the human genome (Thum and Condorelli, 2015; Bar et al., 2016). In addition to transfer RNAs (tRNAs) and ribosomal RNAs (rRNAs), the most well-known ncRNAs are long non-coding RNAs (lncRNAs) and microRNAs (miRNAs or miRs). MiRNAs are short, single-stranded, and highly conserved ncRNAs that widely exist in eukaryotes. MiRNAs are involved in several gene expression regulatory programs including mRNA degradation and translational repression *via* RNA interference (RNAi) mechanisms (Beermann et al., 2016). MiRNAs are mainly transcribed by RNA polymerase II as pri-miR and subsequently processed by the RNase III endonucleases Drosha and Dicer (Cullen, 2004). The mature 18 to 21 nucleotide miRNAs then bind immediately to Argonaute (AGO) proteins to form the RNA-induced silencing complex (RISC). Next, the seed region of the miRNA (normally the first 1–8 nucleotides) binds to the complementary site of the target mRNA which then induces RISC-mediated recruitment of suppression factors to inhibit protein translation. Through association with different proteins of the AGO family, the RISC can also directly degrade targeted mRNAs, while the endonuclease activity is specifically mediated by AGO2 (Bartel, 2009). Several miRNAs have already been identified for their essential roles in heart and kidney disease (Care et al., 2007; Thum et al., 2008; Corsten et al., 2010; Lorenzen et al., 2011; Lorenzen et al., 2013; Lv et al., 2013; Lorenzen et al., 2014; Zan et al., 2014; Zawada et al., 2014; Gaede et al., 2016; Vegter et al., 2016; Wang et al., 2016; Zhang et al., 2016; Rahmel et al., 2018). Many miRNAs are known to be involved to varying degrees in both the acute and chronic phases of primary organ dysfunction in CRS, however, miR-21 specifically has been reported in all types of CRS (**Figure 1** and **Table 1**). Interestingly, miR-21 is highly expressed in the heart and kidneys and elevated levels of miR-21 lead to a poor outcome in most primary organ dysfunctions (Kumarswamy et al., 2011; Du et al., 2013; Zhou et al., 2018). Nevertheless, some studies also showed that miR-21 is a cardio- and reno-protective miRNA in acute disease states, such as acute myocardial infarction. Based on the association and promising preclinical data for antisense oligonucleotide (ASO)-mediated targeting of miR-21 in the heart and kidneys (Thum et al., 2008; Zhong et al., 2011; Liang et al., 2012; Wang et al., 2013; Chuppa et al., 2018; Hinkel et al., 2020), suppression of miR-21 may represent an attractive therapeutic option for the treatment of CRS. In this mini review, we aim to give a concise overview of the role of miR-21 in different clinical manifestations of CRS. In addition, we summarize the current state of oligonucleotide-based drugs.

**Table 1:** Other potential miRNAs that are involved in cardiorenal syndrome.

Type	Primary dysfunctional organ	Secondary dysfunctional organ	Related miRNAs	Reference
1	Acute heart injury	Acute kidney injury	miR-21, miR-208, miR-320	(Dong et al., 2009; Cheng et al., 2010; Corsten et al., 2010; Lorenzen et al., 2013; Vegter et al., 2016; Yang et al., 2018)
2	Chronic heart failure	Chronic kidney disease	miR-21, miR-22, miR-133	(Care et al., 2007; Zawada et al., 2014; Zhang et al., 2016)
3	Acute kidney injury	Cardiovascular disease	miR-21, miR-20a, miR-24	(Thum et al., 2008; Godwin et al., 2010; Lorenzen et al., 2014; Vegter et al., 2016)
4	Chronic kidney disease	Chronic heart failure	miR-21, miR-155, miR-29	(Lv et al., 2013; Gaede et al., 2016; Chuppa et al., 2018)
5	Systemic disease (sepsis, DM, SLE … etc)	Cardiac and renal dysfunction coexist	miR-21, miR122, miR-146a	(Lorenzen et al., 2011; Wang et al., 2016; Rahmel et al., 2018)

DM, diabetes mellitus; SLE, systemic lupus erythematosus

## MiR-21 In Cardiac And Renal Dysfunction

### MiR-21 in Heart Disease

MiR-21 was first identified to be robustly up-regulated in cardiac hypertrophy and was later shown to be a cardiac fibroblast-derived miRNA (**Figure 1**). MiR-21 suppresses *Sprouty homolog 1* (*Spry1)* expression, and thus enhances ERK-MAPK activity which leads to fibroblast activation/proliferation and cardiac fibrosis (Thum et al., 2008). Silencing of miR-21 by antagomir ASOs in the transverse aortic constriction (TAC) mouse model; efficiently blocks the ERK-MAPK signaling pathway, reduces interstitial fibrosis, and restores cardiac function. Interestingly, miR-21-3p, also known as the miR-21 passenger strand or miR-21*, which was thought to be degraded during miRNA biosynthesis, can also regulate cardiac hypertrophy. Bang et al. showed that miR-21-5p (the guide strand) is enriched in cardiac fibroblasts, while miR-21-3p is enriched in fibroblast-derived exosomes (**Figure 1**). By targeting *SORBS2* and *PDLIM5* through paracrine secretion to cardiomyocytes, miR-21 induces cardiac hypertrophy. Inhibition of miR-21-3p expression by an antagomir could reverse this phenotype (Bang et al., 2014). In addition to the modulation of fibrosis signaling pathways, Liang et al. demonstrated that TGF-β1 can directly activate miR-21 expression and further induce cardiac fibrosis through activating collagen and α-SMA protein expression; whereas inhibition of miR-21 reverses these alternative fibrosis pathways (Liang et al., 2012; Lorenzen et al., 2015). Apart from directly inducing fibrosis, miR-21 has also been reported to participate in the endothelial–mesenchymal transition (EndMT), which indicates the multi-functional role of miR-21 in cardiac fibrosis (Kumarswamy et al., 2012; Ghosh et al., 2012).

Nevertheless, in an acute heart injury such as in the early phases of acute myocardial infarction (AMI) or ischemic preconditioning (IP), a temporary injury known to be cardioprotective, miR-21 appears to function differently between acute and chronic heart disease. A study conducted by Dong et al. showed that miR-21 expression decreased in the infarct area of the heart 6 h after AMI and increased 6 h after IP. Overexpression of miR-21 *via* an adenovirus significantly reduces infarct size and cardiomyocyte apoptosis. In contrast, inhibition of miR-21 by ASOs increased cardiomyocyte apoptosis and programmed cell death 4 (PDCD4) protein expression (Dong et al., 2009; Cheng et al., 2010; Yang et al., 2018). This conflicting data indicates that varying conditions need to be taken into account when considering different treatments to target miR-21 such as: different cardiac cell types, the specific type of heart disease, and the state of disease progression.

### MiR-21 in Kidney Disease

Similar to its role in heart disease, miR-21 contributes to the progression of acute and chronic stages in kidney disease and also has distinct roles in early and late disease stage (Hinkel et al., 2020). In some studies, AKI induced by ischemia reperfusion injury (IRI), activates massive inflammation, epithelial cell apoptosis, and fibrosis (Brandenburger et al., 2018). During inflammation, macrophages activate miR-21 expression in renal fibroblasts through two pathways: 1) macrophages secrete high level of interleukin-6 (IL-6) which activate STAT3 and further induce miR-21 expression in renal fibroblasts; 2) macrophages secrete small extra-cellular vesicles enriched with miR-21 that are transferred to renal fibroblasts (Schauerte et al., 2017) (**Figure 1**). These renal miR-21 levels inhibit Notch2 expression which induces increased fibrosis markers such as collagen 1 and α-SMA. Liu et al. also showed that miR-21 directly participates in renal fibrosis by targeting *DDAH1 (* Liu et al., 2016 *)* which is a key gene involved in the pathological process of end-stage AKI. In other cases of ischemia/reperfusion-induced AKI, miR-21 was shown to be reno-protective. Song et al. showed that miR-21 protects the kidney from ischemia/reperfusion-induced AKI by preventing the apoptosis of renal epithelial cells and inhibiting an inflammatory response (Foinquinos et al., 2020). Xu and colleagues also demonstrated that miR-21 was up-regulated after renal IP and attenuated IRI in the kidneys (Lorenzen et al., 2015). Different from AKI, in a mouse model for diabetic nephropathy, miR-21 is mainly expressed in cortical glomerular and tubular cells and induces renal fibrosis which eventually leads to CKD (Wang et al., 2013). Inhibition of miR-21 by antagomir ASOs significantly decreased inflammation; cell apoptosis; and reduced collagen I, α-SMA, and fibronectin expression in a mouse model for diabetic nephropathy (Wang et al., 2013). In addition, TGF-β1 activates miR-21 expression, not only in cardiac fibroblasts, but also in renal tubular epithelial cells (TECs) through Smad3 activation (Zhong et al., 2011). Suppression of Smad3 prevents TGF-β1-induced miR-21 expression, and thus prevents renal fibrosis. Moreover, direct silencing of miR-21 by antimir ASOs reduces fibrosis markers *in vivo* and *in vitro (* Zhong et al., 2011 *)*.

### MiR-21 in Systemic Disease

Inflammation is a common feature of systemic diseases; thus it is not surprising that miR-21 also plays an important role such diseases like sepsis, DM, hypertension, and systemic lupus erythematosus (SLE). In sepsis-induced cardiac dysfunction, Wang et al. showed that miR-21-3p is significantly up-regulated in the heart after lipopolysaccharide (LPS)-induced sepsis in mice. Similar to Bang and colleagues' work, miR-21 altered the cardiac α/βMHC ratio and caused cardiac dysfunction through targeting *SORBS2*, while inhibition of miR-21 by antagomir ASOs improved cardiac function and prevented mitochondrial ultrastructure damage (Wang et al., 2016). In a type II DM mouse model, Zhong and colleagues showed that renal miR-21 expression increased compared to healthy mice. Zhong et al. further demonstrated that miR-21 inhibits *Smad7* expression which results in activation of TGF-β and NF-κB. Knockdown of miR-21 restores *Smad7* expression and ameliorated microalbuminuria, renal fibrosis, and inflammation (Zhong et al., 2013). Hypertension is a major contributor to type 2 and type 4 CRS, Nohria et al. analyzed data from the ESCAPE Trial and found that patients with a history of hypertension and DM were associated with decreased renal function (Nohria et al., 2008). However, using diuretics or ACE inhibitors to treat hypertension, cause side effects, such as hypotension, and therefore, reduced the renal afferent arteriole pressure and glomerular filtration rate (House, 2013). Additionally, Nohria et al. found that improving heart function did not further improve renal function. The current treatments of CRS are limited, and therefore require further research to develop drugs with new targets. Another systemic disease is SLE (or lupus), an autoimmune disease characterized by an excessive immune reaction against self-antigens. Auto-antibodies that are produced by hyperactive B cells and from defects in T cell-dependent B cell activation, are two major causes of SLE (Fernandez-Gutierrez et al., 1998; Dorner et al., 2011). It has been shown that miR-21 is up-regulated in lupus B and T cells, and the silencing of miR-21 with ASOs is able to significantly reverse splenomegaly, one of the clinical manifestations in SLE patients (Garchow et al., 2011; Zan et al., 2014) (**Figure 1**). Similarly, Garchow et al. induced SLE in miR-21 knockout mice and found a reduction of CD28:CD80/86 and CD40:CD154 co-stimulatory pathways which are responsible for initiating an immune response and for T cell activation (Garchow and Kiriakidou, 2016).

## MiRNA Inhibition By Antisense Oligonucleotides

Derailed expression of miRNAs and the subsequent functional consequences may be treated by novel RNA therapeutics. Endogenous downregulation can be compensated with synthetic miRNAs, known as miR-mimics, whereas increased expression can be blocked by single-stranded miRNA-specific antisense molecules. While both strategies have been successfully applied in numerous preclinical studies, the clinical use of miRNA therapeutics is still very challenging. RNAs are extremely sensitive and vulnerable to ribonuclease-mediated degradation, which is present in both cellular and extra cellular environments. These properties do not allow the use of “naked” antisense oligonucleotides (ASOs) *in vivo* since they will soon be degraded by ribonucleases in the bloodstream of the target organ (Layzer et al., 2004; Watts et al., 2008). To circumvent this problem, chemically modified nucleotides are used to stabilize the ASOs. At the same time, certain modifications may also assist with their cellular uptake. In this context, Banks et al. demonstrated that phosphorothioate modified ASOs enter cells through membrane-bound receptors (Banks et al., 2001), however, most unmodified ASOs are taken up by cellular endocytosis (Juliano, 2016). Currently, there are three major types of modifications: 1) Backbone modifications, 2) locked nucleic acids (LNA), and 3) Ribose 2′-OH group modifications (Lam et al., 2015; Sardone et al., 2017).

### Backbone Modifications: Phosphodiester, Phosphorothioate, Boranophosphate

The first generation of modified ASOs are phosphorothioate-modified (PS)-ASOs which are characterized by a substitution of the non-bridged phosphate oxygens with Sulphur atoms. The PS-ASOs are highly resistant to nucleases and have high retention rates in the body due to their minimal clearance by the kidney and urinary excretion. However, a negative side effect is that PS-ASOs can also bind non-specifically to proteins, and thus may cause cytotoxicity (Gura, 1995; Khaled et al., 1996).

### Locked Nucleic Acids

Through a methylene bridge between the 2′-O and the 4′-C of the ribose, LNA-ASOs have a stable “locked” 3′-endo-conformation structure which allows them to be nuclease resistant. With this “locked” structure, LNA-ASOs can allow for more stable and much more specific binding to a target RNA. Notably, the pure LNA-ASO structure is not able to induce robust RNase-mediated cleavage of the ASO-miRNA hybrid, but instead, blocks the miRNA from binding to its target. To circumvent this issue, a short stretch of DNA (6–8 nucleotides) can by placed between two LNA structures (Kurreck et al., 2002). Such DNA/LNA mix-mers, called “GapmeR” form a short DNA-RNA hybrid between the short DNA and the targeted RNA which induces efficient cleavage by RNase H. Both single LNA- and GapmeR-modified ASOs could be used to inhibit miRNA expression. One can also combine two modifications to form an LNA-GapmeR ASO for the inhibition of most mRNAs, lncRNAs, and miRNAs (Kurreck et al., 2002; Elmen et al., 2008).

### Ribose 2′-OH Group: (2′-O-Me, 2′-F, 2′-O-MOE)

A ribose 2′-OH group modification is the second generation and best studied type of ASOs. By substituting the ribose 2′-OH group with other groups, such as 2′-O-methyl (2′-O-Me), 2′-fluoro (2′-F), and 2′-methoxyethyl (2′-O-MOE), the ribose-ASOs gain nuclease resistance, enhanced stability, and an increased half-life *in vivo*. In addition to nuclease resistance, ribose-ASOs have other advantages including higher specificity to their targeted RNA and reduced immune activation (Judge et al., 2006; Elmen et al., 2008).

Importantly, all the modification mentioned above can also be combined to further enhance stability and specificity. For example, the miRNA inhibitor “antagomir” is an ASO that is modified with 2′-O-Me, PS, and a cholesterol group, which lends them better nuclease resistance, high specificity, and enhanced cellular uptake efficiency (Krutzfeldt et al., 2005; Lennox et al., 2013). Currently there are several ASOs in Phase I/II clinical trials (Gallant-Behm et al., 2018; Seto et al., 2018) (**Table 2**), among them are Miravirsen and RG-012, which specifically target miR-122 and miR-21, respectively. MiR-122 is a liver-specific miRNA that is essential for hepatitis C virus (HCV) accumulation and replication (Jopling, 2008). *In vitro* and *in vivo* treatment with Miravirsen, an ASO with both LNA and PS modifications, showed significant down-regulation of miR-122, reduction of interferon-regulated genes, and improvement of HCV-induced liver pathology (Lanford et al., 2010; Gebert et al., 2014). RG-012, which is modified with PS and 2′-O-MOE, is currently being tested as a cure for Alport syndrome. Alport syndrome is a kidney disease caused by mutations in collagen genes (*Col4A3, Col4A4*, and *Col4A5*). The basement membranes are assembled with collagen type IV, laminins, and proteoglycans. Six different genes (from *Col4A1* to *Col4A6*) encode six chains of collagen type IV (collagen IV α1 to α6), and of them, three chains (e.g. α3, α4, and α5) are assembled into one protomer. However, in Alport syndrome, the genes responsible for encoding collagen type IV are mutated. For example, the major mutation for the *Col4A5* gene is on chromosome Xq26–48, and for the *Col4A3* and *Col4A4* genes, the mutation is located on chromosome 2q35–37. These mutations prevent proper protomer assembly and, therefore, result in a defective glomerular basement membrane and kidney dysfunction (Hudson et al., 2003). Patients of Alport syndrome suffer from kidney dysfunction, hearing loss, and eye abnormalities. Administration of RG-012 to inhibit miR-21 leads to a slowed progression of renal fibrosis and an increased lifespan in Alport syndrome mouse models (Gomez et al., 2015).

**Table 2:** Current ASOs in clinical trials.

Drug	Target	Indication	Chemical modification	Phase	Reference
**RG-012**	miR-21	Alport syndrome	2′-O-MOE, PS	I/II	(Gomez et al., 2015)
**Miravirsen (SPC3649)**	miR-122	Hepatitis C virus	LNA, PS	ll	(Lanford et al., 2010; Gebert et al., 2014)
**RG-101**	miR-122	Hepatitis C virus	GalNAc	Terminated, metabolic/hepatic toxicity	(van der Ree et al., 2017)
**Cobomarsen (MRG-106)**	miR-155	CTCL (MF subtype)	LNA	I/II	(Seto et al., 2018)
**S95010 (MRG-110)**	miR-92	Accelerate wound healing	LNA	l	(Gallant-Behm et al., 2018)

CTCL, cutaneous T-cell lymphoma; MF, mycosis fungoides

## Challenges In Aso Therapy

Despite advances in development and therapeutic applicability, several challenges for ASO drugs still need to be solved (Frazier, 2015; van der Ree et al., 2017) (**Table 3**). One challenge is to reduce the potential off-target effects of ASOs, but also to regulate undesired on-target effects that may represent an issue. For example, miR-21 is not organ or cell type-specific and has distinct functions depending on the organ and/or disease progression. Thus, a patient may suffer from undesired effects of miR-21 ASO therapy in one organ (e.g. the heart) despite successfully targeting of miR-21 in another organ (e.g. the kidney). This may even be the case in the very same organ where antisense treatment may be beneficial for one cell type (e.g. cardiomyocytes) and at the same time detrimental in another cell type (e.g. endothelial cells). To reduce off-target effects, careful dose-regulation studies are required to determine the optimal concentration (Shen and Corey, 2018). In addition, bioinformatics analysis for optimal and specific sequence design is another way to improve ASO specificity, although due the short sequences of miRNAs, the options are very limited in comparison to mRNAs or lncRNAs. The length of ASOs is another important factor for reducing off-target effects. Yoshida et al. found that ASOs with at least 13-mer in length show a higher specificity. Thus, it is conceivable that that the shorter the ASO, the higher the number of complementary regions for unintended off-target RNAs (Yoshida et al., 2018). For instance, Miravirsen is a 15-mer ASO which is sufficiently long enough to minimize the potential off-target effects. A further challenge is immunostimulatory effects that can be caused by the chemical modifications of ASOs. It has been shown that a 2′-O-MOE modification induces pro-inflammatory responses including elevated cytokine levels and immune cell infiltration in the liver (Senn et al., 2005). Moreover, potential hepatic and renal toxicity are known issues. Burdick et al. showed that LNA-ASOs with certain sequence motifs (TCC and TGC), cause hepatotoxicity indicated by liver lesions (Burdick et al., 2014). Renal toxicity such as glomerulopathy has also been reported in some cases; however, the control group in these studies is phosphate buffer saline (PBS) as opposed to ASO-scramble which did not explain whether the renal toxicity arose from ASO-mediated gene inhibition or the structure/chemical modification of the ASO itself. Therefore, the relationship between renal toxicity and ASOs is still unclear and warrants further investigation (Herrington et al., 2011; Frazier et al., 2014). A recent study assessed 11 different ASOs that had been tested in 32 clinical trials; here the authors found no evidence for renal dysfunction in ASO-treated patients (Crooke et al., 2018). Nevertheless, hepatic and renal functions should be closely monitored, especially when giving ASO drugs to CRS patients. Finally, the delivery of the ASO drug to disease-specific sites also represents big hurdles for RNA-based therapeutic strategies. The systemic delivery of ASOs does not allow for exclusive targeting of the diseased organ, while organ-specific delivery requires invasive procedures. In the same context, despite the various modifications of ASOs, their often-inefficient penetration of the cell membrane's lipid bilayer remains an issue. To achieve better cellular uptake, there are two major delivery systems for ASOs under intense investigation which involve the packaging of ASOs into polymer- and lipid-based nanoparticles (Layzer et al., 2004; Watts et al., 2008). Furthermore, ASOs may also be delivered through conjugation with antibodies (Lu et al., 2013). In particular the latter seems to have a good clinical prospect given that cell surface-specific antigens for the target cell type can be identified.

**Table 3:** Possible side effects and solutions in ASO therapy.

Side effects	Cause	Solutions
**Off-target**	Organ non-specific	Organ specific delivery method
		Dosage test to find optimal concentration
	Non-specific binding	Improve bioinformatics analysis
	ASO with short sequence	Increase the length to 13 to 15-mer
**Immunostimulatory**	Chemical modification	Avoid the known modifications that cause immunostimulation
**Organ toxicity**	Toxic motif (TCC/TGC)	Avoid toxic motif when designing ASO

Current treatments for CRS are mainly small molecule drugs. ASOs that specifically inhibit miR-21 expression could be promising drugs for CRS through attenuating cardiac and renal fibrosis. Potential off-target, inflammatory response, and hepatic/renal toxicity of ASOs are the main challenges in an ASO-based therapy and need to be monitored carefully.

## Summary and Outlook

With an increased level of concrete preclinical evidence, more clinical trials for ASO-based therapies are ongoing and several ASO-based drugs are already FDA approved (Stein and Castanotto, 2017). Here, we summarized the current knowledge on the role of miR-21 mostly in primary dysfunctional organs, such as the heart and the kidneys. However, since both organs are affected in complex and not yet fully understood mechanisms in CRS, more experiments need to be performed in an appropriate and practical CRS model. Chuppa et al. demonstrated that suppression of miR-21 improved cardiac function in type 4 CRS (Chuppa et al., 2018) and Rana et al. showed that miR-21 expression positively correlated with uremic toxin levels (urea, creatinine, phenols etc.) in type 2 CRS. Treatment with AST-120, an orally administered intestinal sorbent that lowers serum uremic toxins, attenuated cardiac fibrosis and down-regulated miR-21 expression. An ASO targeting miR-21 taken with AST-120 again reinforces the notion that miR-21 may be a promising therapeutic target in the treatment of CRS (Rana et al., 2015). Recently, a treatment of antimiR-132 in patients with heart failure, which is a trigger for type 2 CRS, also went into first-in-human trials (NCT04045405) (Foinquinos et al., 2020). With the remarkable progress in genome sequencing, personalized medicine, and gene and ASO therapies, there are now novel innovative therapeutic strategies available that add up to the repertoire of traditional small molecule or protein based drugs. Nevertheless, continuous research is needed to further improve ASO chemical modifications, target specificity, and to determine optimal delivery routes and drug dosing. With this in mind, we are hopeful that more ASO-based drugs will enter the market in the near future, eventually including those that could treat CRS.

## Author Contributions

CK-H and CB prepared the manuscript. CK-H prepared figures. TT revised the manuscript.

## Funding

This work was supported by the Federal Ministry of Education andResearch (BMBF, research grants ERA-CVD JTC2016 EXPERT, 01KL1711 and ERA-CVD JTC2018 INNOVATION, 01KL1903) and the German Research Foundation (DFG,Projects TH903/18-1, TH903/20-2, and BA5631/2-1).

## Conflict of Interest

TT has filed and licensed patents about the diagnostic and therapeutic use of several cardiovascular microRNAs. TT is founder and shareholder of Cardior Pharmaceuticals GmbH.

The remaining authors declare that the research was conducted in the absence of any commercial or financial relationships that could be construed as a potential conflict of interest.
